# Physical Training In-Game Metrics for Cognitive Assessment: Evidence from Extended Trials with the Fitforall Exergaming Platform

**DOI:** 10.3390/s21175756

**Published:** 2021-08-26

**Authors:** Evdokimos I. Konstantinidis, Panagiotis D. Bamidis, Antonis Billis, Panagiotis Kartsidis, Despoina Petsani, Sokratis G. Papageorgiou

**Affiliations:** 1 Laboratory of Medical Physics and Digital Innovation, School of Medicine, Aristotle University of Thessaloniki, 54124 Thessaloniki, Greece; pdbamidis@gmail.com (P.D.B.); antonis.mpillis@gmail.com (A.B.); panos.kartsidis@gmail.com (P.K.); despoinapets@gmail.com (D.P.); 2 Memory Disorders and Rare Dementias Unit, 1st Department of Neurology, Eginiteion University Hospital, National and Kapodistrian University of Athens, 15772 Athens, Greece; sokpapa@med.uoa.gr

**Keywords:** assistive technologies, clinical decision-making, exergames, in-game metrics, serious games

## Abstract

Conventional clinical cognitive assessment has its limitations, as evidenced by the environmental shortcomings of various neuropsychological tests conducted away from an older person’s everyday environment. Recent research activities have focused on transferring screening tests to computerized forms, as well as on developing short screening tests for screening large populations for cognitive impairment. The purpose of this study was to present an exergaming platform, which was widely trialed (116 participants) to collect in-game metrics (built-in game performance measures). The potential correlation between in-game metrics and cognition was investigated in-depth by scrutinizing different in-game metrics. The predictive value of high-resolution monitoring games was assessed by correlating it with classical neuropsychological tests; the area under the curve (AUC) in the receiver operating characteristic (ROC) analysis was calculated to determine the sensitivity and specificity of the method for detecting mild cognitive impairment (MCI). Classification accuracy was calculated to be 73.53% when distinguishing between MCI and normal subjects, and 70.69% when subjects with mild dementia were also involved. The results revealed evidence that careful design of serious games, with respect to in-game metrics, could potentially contribute to the early and unobtrusive detection of cognitive decline.

## 1. Introduction

Conventional clinical cognitive assessment is not part of the older adult’s everyday life [1] and usually only takes place when the patient or family has concerns regarding cognitive dysfunction [2]. Moreover, the clinical environment visit increases stress, which may in turn affect negatively the assessment [3] or act as amotivation for the patient to perform well on the tests [4] to avoid stigmatization. These factors contribute to questioning the ecological validity of neurophysiological tests [5] and may lead to delayed detection, or failure to detect cognitive decline, or even to false diagnosis among primary care providers [3,6]. The need for fast and cheap screening tests [7] with good discriminant capacity, even when distinguishing between various degrees of cognitive impairment [8], has led recent research ventures to look for alternatives to the paper and pencil screening tests that are more acceptable for older adults [9].

Game-like applications designed for a primary purpose other than pure entertainment [10,11] and virtual reality (VR) [12], following Plato’s statement that “...you can discover more about a person in an hour of play than in a year of conversation ...”, are generating strong interest among the research community in the use of serious games (SGs) as psychometric tools and indicators [13]. SGs for older adults are considered to have the potential to provide more reliable information in terms of assessment compared to conventional methods [13,14], since users (i.e., the persons playing the games) do not perceive the SG as a stressful testing procedure [15]. SGs for older adults have recently been categorized either as preventive and therapeutic or as assessment-oriented [16]. Cognitive measures in game-like interfaces contribute to the early detection of neurological disease [17], while exergames [18] (serious games focusing on engaging users in physical activity or exercise through the games) are presented as promising tools for measuring and assessing physical health unobtrusively [18,19].The latter focus mainly on fall risk assessment by correlating typical in-game metrics of exergames, such as movement time and response time, with a test battery of standardized assessment tests of fall risk [20].

Although SGs have been utilized for cognitive assessment for some time [21,22], exergames have only recently been introduced into this domain. Only recent studies exhibit correlations between exergames’ performance features with cognitive assessment tests, and between in-game metrics with neuropsychological tests, including MMSE [23,24,25]. SGs can shape stealth assessment [26] when they are utilized as formative assessment tools (continuously monitoring throughout the game intervention) [14], incorporating the assessment process unobtrusively in the intervention process. Such a combination moves SGs beyond focusing merely on intervention or screening, leading to a dual-role SG where intervention per se is supported by continuous assessment. However, it is necessary to address the risk of investing in technical features that could potentially affect the reliability of the game, thus intertwining the purpose of enhancing a feature with that of its measurement [27].

We postulate herein that unobtrusive data gathering could be considered as an untapped potential of exergames along with their intervention role. Built-in performance measures could be efficient, cognition specific, cost-effective and time-saving [19] in distinguishing between cognitively healthy older adults and those with mild cognitive impairment. Using long-term data from unobtrusive monitoring via computer games can be exploited for the detection of deterioration trends in cognitive performance beyond one shot screening tests/games with test–retest constraints. Moreover, the unobtrusive detection of changes in the cognitive baseline through SGs may address the gaps in clinical assessment [4]. The rationale behind this argument may lie in the fact that games motivate the patient to participate for enjoyment, thereby eliminating the stress induced by clinical assessment tests.

The platform has been used as a physical exercise intervention tool by older adults, following the recommendations for physical activity and public health in older adults from the American College of Sports Medicine and the American Heart Association [28].The purpose of this study was to investigate the potential value of an exergaming platform, with evidence-based findings [18], as an assessment tool as well as an exercise device, without comparing it with SGs, which target only cognitive assessment. This platform collects unobtrusive measurements during the activity; these are the so-called in-game metrics. The potential predictive value of the in-game metrics was assessed by (i) correlating them with classical cognitive screening tests, such as the MMSE and MOCA, and (ii) estimating sensitivity and specificity in detecting MCI by measuring the area under the curve in the receiver operating characteristic based on the clinical diagnosis of a dementia expert neurologist.

## 2. Materials and Methods

FitForAll (FFA) [18] is an exercise-based, serious game blended (exergaming) platform, initially relying on the Nintendo Wii Remote and Balance Board controllers in order to detect the user’s motion, posture and gestures. It consists of carefully designed games aimed at older adults’ physical exercise and the maintenance/advancement of a healthy physical status and wellbeing. Focusing predominantly on appropriate physical training, the physical exercise objectives rely on specific guidelines from the American College of Sports Medicine and American Heart Association [28]. The full game suite is composed of aerobic, resistance, flexibility and balance computerized exercises administered in a gamified way.

### 2.1. Intervention and Monitoring Games

The combination of games promoting physical exercise (aerobic, resistance, flexibility and balance) in an ordered sequence instantiates a physical training “session” which may stand on its own or be part of a whole intervention protocol. During the resistance and flexibility exercises, the users follow the instructions provided on the screen while a picture of positive valence is revealed gradually after each successful repetition. The balance exercise games make use of a color code and virtual footprints on the screen, guiding the user to specific movements. During aerobic exercises, the user’s avatar moves through a city landscape to render the exercise enjoyable.

FFA also incorporates a set of high-resolution monitoring games (HRMG) that require a combination of physical and light cognitive effort in order to be accomplished. The required cognitive functions implicated in the games include simple and choice reaction, concentration, perception, learning and memory, visuospatial coordination, visuomotor tracking, divided attention, cognitive flexibility and processing speed.

The HRMG include five games: Ski Jump, Apple Tree, Arkanoid, Fishing and Mini Golf. In Ski Jump, the users control the avatar’s jump by moving the center of mass to a specific position. In the Apple Tree game, users control a basket picking apples from a tree by moving their center of mass. Similarly, in the Arkanoid game users control the horizontal position of a bar and attempt to hit a moving ball, while in the Fishing game older adults control the vertical position of a boat while attempting to catch the horizontally moving fishes. In Mini Golf, users move their center of mass on the balance board and attempt to put a ball into a hole by overcoming different barriers.

### 2.2. Difficulty and Exertion Management

The FFA training protocol is divided into 4 difficulty levels [18] to accommodate the participants’ fitness level improvements [28], following the recommendations for keeping users in the “flow zone” which represents the feeling of complete and energized focus on an activity with a high level of enjoyment and fulfilment [13]. Older adults start from the lower difficulty level and are promoted to the appropriate level according to their performance on a periodically administered Fullerton Fitness Test [29]. Fatigue management in SGs is handled by the alternation of physically intense and less challenging game periods, allowing players to relax and recover [30].

### 2.3. FitForAll In-Game Metrics

The majority of the games measure the correctly accomplished tasks or repetitions within a specific time as a score. “Correctly” is defined in terms of the required movement range—degrees, steps, etc. The HRMG metrics rely on the total completion time as well as the number of missed or gathered points/targets, the degree of deviation from the optimal path, the achieved goal and the number of attempts required for goal accomplishment (Table 1). The specific coefficients for the score calculations were determined in collaboration between a statistician and the physical exercise expert contributing to the design of the games, to provide a smooth distribution of scores. Objective measurements were also integrated by recording systolic/diastolic pressure and heart rate, especially after intensive exercises (manually measured by the user). On the subjective metrics axis, older adults were asked to communicate their perceived fatigue level through a graphic representation of the Borg rating of perceived exertion scale [31].

### 2.4. Study’s Features Based on FitForAll In-Game Metrics

Each game’s score, normalized on a 10-point scale, was calculated by the value of the metrics monitored during each game, as presented in Table 1. The factors in the equations in Table 1 were set based on expert opinion. According to these individual scores, an aggregated score per exercise domain (aerobic, resistance, flexibility and HRMG) was calculated for each session. The same approach was followed for vital signs and the Borg scale, where the mean value of the measures was calculated per session. The Borg scale rating of perceived exertion is a widely used and reliable indicator to monitor and guide exercise intensity. The mean value, the slope and the intercept were calculated for each session of each type of exercise at each level of difficulty, following the equation: y = ax + b (a: slope, b: intercept). As a result, the mean, slope and intercept values for the total training period and the 4 levels of difficulty were extracted and used as features for the analysis. The slope value (first order derivative) for each training period at a specific difficulty level represented performance change speed. A higher slope indicated better performance from session to session (on average) within a training period at a specific difficulty level.

### 2.5. Intervention

The FFA platform was the Physical Training Component in the Long Lasting Memories (LLM) project funded by EU [32]. During the LLM trials, each user had to undergo a 1 h physical training protocol consisting of sessions of 20 min aerobic and 10 min flexibility exercises, 8–10 resistance exercises and 2 balance-targeted exercises (in compliance with the recommendations for physical activity and public health in older adults from the American College of Sports Medicine and the American Heart Association [28]), as well as the HRMG. The intervention was organized in groups of 3–12 older adults under formal carer supervision. Each carer supported the participants to navigate through the screens to the next game and to use the right fitness equipment, as well as to measure their blood pressure and heart rate when required. The latter occurred every ~10 min, especially after intensive aerobic exercise, allowing a break of 2–3 min. Our previous study proved the effectiveness of the intervention by demonstrating statistically significant improvement in lower and upper body strength and flexibility, aerobic endurance and dynamic balance [18]. Based on the carers’ observations, their workloads were diminished after 4–6 sessions. The adherence level (the proportion of sessions attended by FFA participants with respect to the planned sessions) reached a level of 82% [18]. The trials were conducted in an environmentally valid manner in numerous settings in Thessaloniki and Athens (Greece), including day care centers of the Greek Association of Alzheimer’s Disease and Related Disorders, municipal social care centers, other senior centers and local parish community centers.

### 2.6. Participants

During the LLM trial period [18], 38 cognitively normal (CN), 64 mild cognitive impairment (MCI) and 14 mild dementia (MD) users were involved in the Thessaloniki-based trials (116 participants). Flyers, workshops, presentations by the team, professional contacts in intervention and associated institutions, advertisement in the local newspapers and word of mouth were all aspects of the recruitment strategy [32]. Inclusion criteria were age ≥55 years with fluent language skills, no severe cognitive impairment, agreement of a medical doctor and time commitment to study period. Exclusion criteria were participation in another study during the same period, unrecovered neurological disorders (i.e., stroke, traumatic brain injury, etc.), physical or psychological disorders preventing participation in the intervention (i.e., inability to follow instructions), unstable medication within the past 3 months, severe and uncorrectable vision loss or wearing a hearing aid for fewer than 3 months [32]. These older adults engaged with FFA for a minimum of 3–4 sessions per week for a total period of 7–8 weeks. No financial incentive was provided to participants and the training program was provided at no cost.

### 2.7. Neuropsychological Examination

A set of tests assessing cognitive status and other specific cognitive domains (attention, memory, executive and visuospatial functions, independent living, etc.) composed the neuropsychological examination that contributed to the diagnostic procedure. All these tests were administrated in their Greek versions: Mini Mental State Examination MMSE [33], Montreal Cognitive Assessment, MoCA [34] and the Trail Making Test (TMT), part B [35]. TMT was used to test cognitive processing and executive functioning. Given the test–retest reliability limitation of MMSE and MOCA, the neuropsychological examination took place 1–2 weeks before the intervention and 1–2 weeks after the intervention (pre–post assessment). A detailed description of the neuropsychological examination may be found in a study by our group [32].

### 2.8. Clinical Diagnosis of Participants

A dementia expert neurologist performed the diagnosis of each participant based on clinical, neuropsychological examination and full laboratory and imaging tests. The diagnosis of Alzheimer’s disease (AD) was given according to criteria outlined by the DSM-IV and the National Institute of Neurological and Communicative Disorders and Alzheimer’s disease and Related Disorders (NINCDS–ADRDA) [36]. Petersen’s criteria [37] were used for the diagnosis of MCI. All participants went through the clinical diagnosis, as it served as the basis for the classification analysis.

### 2.9. Data Analysis

Non-parametric Kruskal–Wallis was chosen for the statistical hypotheses among the games’ scores with respect to the clinical diagnosis, since the majority of variables were not normally distributed (Kolmogorov–Smirnov *p* < 0.05). Significance values were adjusted using the Bonferroni correction for multiple comparisons. Pearson correlations were tested between neurophysiological assessment tests and HRMG scores as they normally distributed (Shapiro–Wilk *p* > 0.05). Finally, both feature selection and classification performed in this study using the multilayer perceptron, a class of feedforward artificial neural network consisting of, at least, three layers of nodes, were conducted through the Waikato Environment for Knowledge Analysis (WEKA). In order to assess the predictive value of the HRMG, the area under the curve (AUC) in the receiver operating characteristic (ROC) analysis was calculated to determine the sensitivity and specificity of the method for detecting MCI based on the clinical diagnosis of the dementia expert neurologist. The ROC of the MMSE and MOCA were also calculated, for comparison purposes, by using the corresponding cut-off scores for MCI.

## 3. Results

Demographics, cognitive assessment scoring and game baseline scores for all groups are presented in Table 2.

### 3.1. Statistically Significant Differences

**Figure 1:**
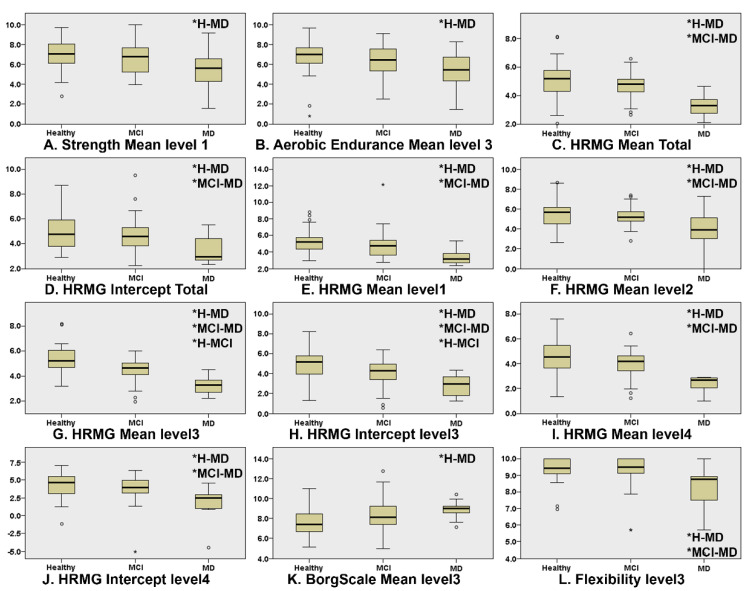
Boxplots of the features where there was statistically significant differences in at least one of the group couple comparisons, namely, CN–MCI, CN–MD and MCI–MD. Independent samples of Kruskal–Wallis were used throughout. HRMG stands for the High-Resolution Monitoring Games, Borg scale is the rating of perceived exertion, strength, aerobic and flexibility represent the scores of the corresponding physical exercises. (* indicates which groups significantly differ from each other).

Figure 1 presents the boxplots of the game scores that exhibited significant differences between at least two of the three cognitive groups (* indicates which groups significantly differ from each other). “Level” corresponds to the difficulty level (lower level numbers indicate less difficulty). The Kruskal–Wallis omnibus comparisons revealed differences between the three groups in Strength Mean level1 (*p* = 0.03, ε2 = 0.073), Aerobic Endurance Mean Level3 (*p* = 0.04, ε2 = 0.054), Borg Scale Mean level3 (*p* = 0.007, ε2 = 0.081), Flexibility Mean level3 (*p* = 0.003, ε2 = 0.141), HRMG Mean Total (*p* = 0.000, ε2 = 0.249), HRMG Intercept Total (*p* = 0.002, ε2 = 0.113), HRMG Mean level1 (*p* = 0.000, ε2 = 0.165), HRMG Mean level 2 (*p* = 0.001, ε2 = 0.149), HRMG Mean Level3 (*p* = 0.000, ε2 = 0.337), HRMG Intercept level3 (*p* = 0.000, ε2 = 0.193), HRMG Mean level 4 (*p* = 0.000, ε2 = 0.240) and HRMG Intercept level4 (*p* = 0.002, ε2 = 0.130). The Kruskal–Wallis pairwise comparisons showed significant differences (*p* < 0.05) between CN and MD in the in-game metrics: Strength Mean level1 (*p* = 0.025), Aerobic Endurance Mean level3 (*p* = 0.035) and Borg Scale Mean level3 (*p* = 0.008) scores. Flexibility Mean level3 (*p* = 0.010) and the vast majority of the HRMG scores presented significant differences not only between CN and MD (Mean Total *p* < 0.001, Intercept Total *p* = 0.001, Mean level1 *p* < 0.001, Mean level2 *p* = 0.001, Mean level3 *p* < 0.001, Intercept level3 *p* < 0.001, Mean level4 *p* < 0.001, Intercept level4 *p* = 0.002), but also between MCI and MD (Mean Total *p* < 0.001, Intercept Total *p* = 0.013, Mean level1 *p* = 0.001, Mean level2 *p* = 0.006, Mean level3 *p* < 0.001, Intercept level3 *p* < 0.006, Mean level4 *p* < 0.001, Intercept level4 *p* = 0.006). Finally, the scores of the HRMG at mean level3 (*p* = 0.004) and intercept level3 (*p* < 0.024) showed significant differences among all group couple comparisons. No statistically significant differences were found for the slope values among any of the three cognitive groups.

### 3.2. Correlation between Metrics and Cognitive Assessments

**Figure 2:**
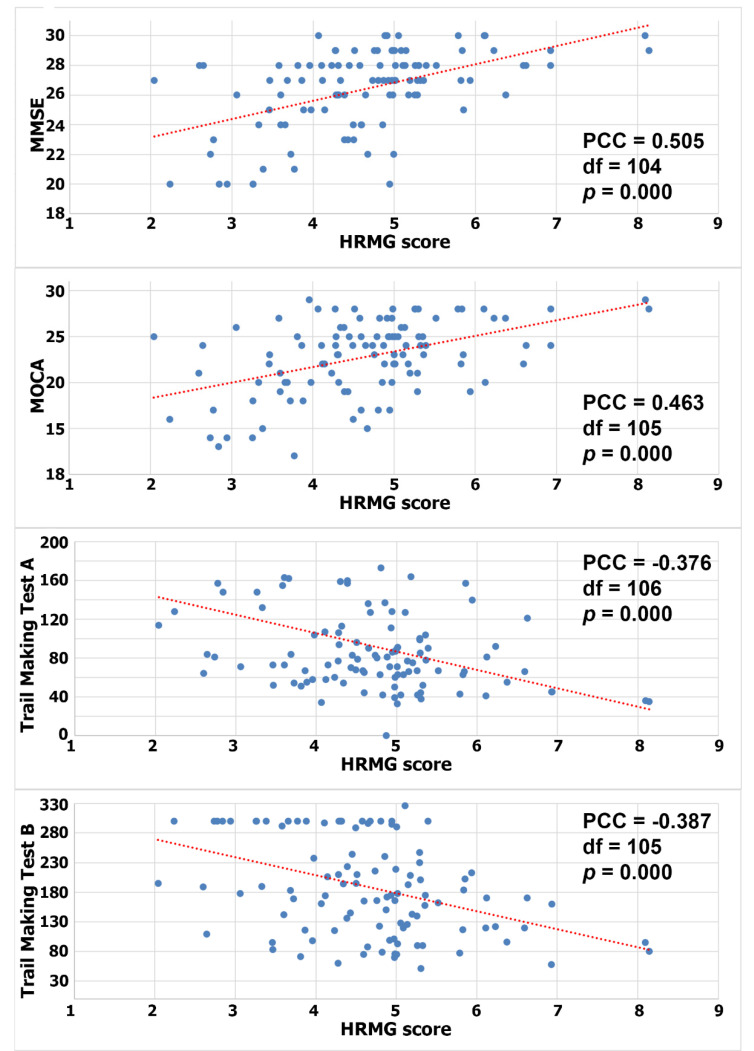
Correlation between High Resolution Monitoring Games and MMSE/MOCA/Trail Making A/Trail Making B, respectively (PCC: Pearson correlation coefficient).

Since the HRMG scores were normally distributed (Shapiro–Wilk *p* > 0.05), Pearson correlations were calculated to test for a linear relationship between HRMG and MMSE, MOCA and TMT A and B (c.f. Figure 2). The correlation between HRMG scores and MMSE and MoCA was moderate (Pearson correlation coefficient 0.505, *p* < 0.005 and Pearson correlation coefficient 0.463, *p* < 0.005 respectively). Similarly, the analyses for correlation between HRMG scores and TMT A and B scores showed modest strength and negative correlations (Pearson correlation coefficient −0.376, *p* < 0.005 and Pearson correlation coefficient −0.387, *p* < 0.005 respectively). The Trail Making Test unit, which is time based, justified the negative correlation, since higher scores indicated poorer cognitive function. No statistically significant linear relationship was found between the slope values and any of the cognitive assessment tests.

### 3.3. Classification of Healthy and Non-Healthy according to In-Game Metrics

The feature selection for the classification procedure was based on the CfsSubsetEval attribute evaluator, which evaluates the worth of a subset of attributes by assessing the individual predictive ability of each feature. A subset highly correlated with the class features, having at the same time low intercorrelation, was preferred. The BestFirst search method searched for attribute subsets by greedy hill climbing augmented with a backtracking facility, both of which were implemented by the WEKA tool. Three clinical diagnosis classes, namely, CN, MCI and MD were considered for the classification procedure. The evaluator ranked Age, HRMG MeanTotal, HRMG InterceptTotal, HRMG MeanLevel1, HRMG MeanLevel3, HRMG MeanLevel4 and HeartRateSlopelevel3 as major features. The multilayer feedforward neural network, an interconnection of perceptrons in which data and calculations flow in a single direction from the input data to the outputs, achieved a classification of 70.69% among CN, MCI and MD. The classification was performed by means of a tenfold cross validation. The overall accuracy was 70.69%. The detailed accuracy for each cognitive status, along with the sensitivity and specificity and the area under the curve (AUC), is presented in Table 3.

### 3.4. Discriminative Validity of HRMG of Cognitively Normal and MCI

**Figure 3:**
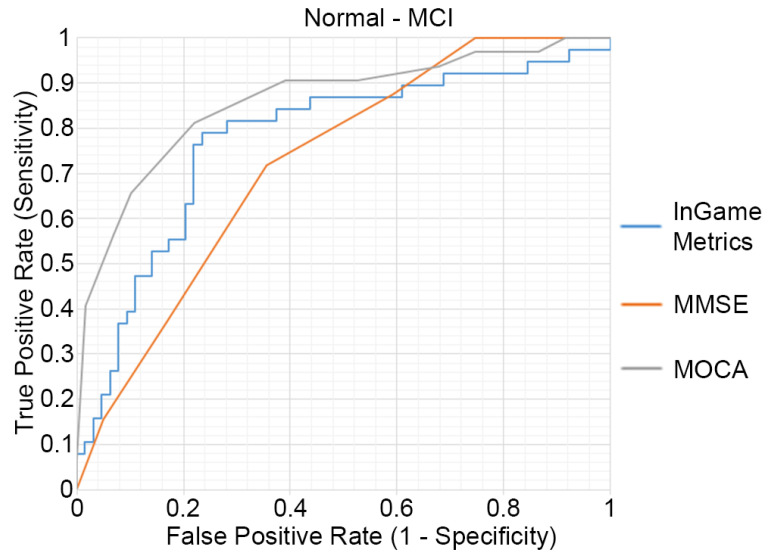
Receiver operating characteristic (ROC) curves of the HRMG metrics, MMSE and MOCA. MCI (n = 64) versus cognitively normal group (n = 38).

The outcomes of the ROC analysis, measuring the abilities of HRMG, MMSE and MOCA to discriminate MCI (N = 64) from cognitively normal (N = 38) older adults, are presented in Figure 3. The HRMG algorithm classified correctly 24/38 cognitively normal and 54/64 MCI subjects. An overall 73.53% classification accuracy was achieved with a maximum AUC of 0.774. Respectively, the AUC for MMSE and MOCA were 0.724 and 0.860.

## 4. Discussion

The study presented in this paper was the first step towards providing evidence through large scale pilots [18] regarding the association of cognitive status with performance in older adults (SG metrics) during exergames. According to the results in this paper, in-game metrics of FFA could classify CN, MCI and MD with an accuracy of 70.69%. The sample size in conjunction with the rigorous intervention program (~2 months), justifies generalization of the potential contribution of exergaming interventions to unobtrusive monitoring of cognitive status through time.

The current study revealed that some game features seemed to discriminate between CN and MD, while the majority also discriminated between MCI and MD. However, only HRMG features at difficulty level 3 discriminated between CN and MCI. This could be attributed to the fact that, as observed by the pilots’ facilitators, this level was neither trivial nor intensive for the average older adult, keeping them in the “flow zone”. In general, the participants considered physical exercise through exergames as light exercise (Borg Scale rating), while the average heart rate (~70 bpm) was close to the target heart rate zone (50–85% of maximum heart rate, 75–127 bpm) [38].

Statistical analysis revealed significant positive moderate correlation between HRMGs and MMSE and MOCA, as well as modest correlation compared to TMT A and TMT B. Previous works in the field, exhibiting lower levels of correlation between game metrics and MMSE, utilized virtual environments while participants walked on a treadmill, attempting to accomplish daily tasks [23]. However, such exercises were not performed by the participants on a daily basis; therefore, they were considered more as screening methods rather than daily intervention and assessment tools. Similar studies focusing only on the cognitive assessment axis and not on interventions in the physical domain [24] exhibit very promising results in identifying MCI patients. The accuracy levels achieved by the classifier, as well as the sensitivity, specificity and AUC when distinguishing MCI subjects from cognitively normal individuals, were comparable to MMSE and MOCA. This must be considered in the light of applying the algorithm to subjects with borderline cognitive decline performance. These results are consistent with the concerns of Vemuri et al. [39] who identified the need for real clinical value for participants whose cognitive health is not clearly defined.

In the light of the absence of an effective/gold standard treatment for dementia, early administration of any available treatment/interventions may be more effective [32] as they may slow cognitive decline [3], thereby improving the quality of the patient’s life [21]. Consequently, a noninvasive, and ideally unobtrusive, low-cost tool that could contribute to early diagnosis and enable regular screening would be a significant ally against cognitive decline and dementia. Furthermore, both the interventional and assessment functions of serious games as presented by FitForAll in this study could potentially be used by older adults themselves without supervision in their home environment. This may have positive effects in two ways. Firstly, they could provide an appropriate ecologically valid environment where diagnostic processes in the form of exergaming could be completely unobtrusive and therefore more valid. Secondly, insurance and public healthcare system costs would be much reduced [13]. However, the key requirement for the effectiveness of SGs, either as intervention or monitoring tools, is engagement with the game. The current study demonstrated high levels of engagement for a period of 7–8 weeks, but available frameworks [40] that could be applied to increase the engagement levels towards measuring performance over a longer period should be taken into consideration during design.

The challenge presented by the large quantity of data gathered by a computer game, beyond the obvious metrics of score and completion percentage [41], is to find ways to access, analyze and understand this wealth of data [42]. Ideally, the game’s data, produced by stealth assessment, could be incorporated into diagnostic systems; better yet, games could be developed as integral components of treatments and interventions, thereby updating the contemporary arsenal of trial/intervention outcome measures. It is believed that once the usefulness of such data is realized, the next logical step would be the maximization of the value of these data by applying data mining and analytics methodologies [42,43].

### Limitations

Despite these important findings, some limitations of this work need to be outlined. FitForAll was primarily designed as an intervention tool, and secondly as an assessment tool. Therefore, in-game metrics were not exploited to the extent warranted. Although the results were promising and constituted evidence that exergames could contribute to the early detection of cognitive decline, further research and wider pilots in terms of participants and duration would give a clearer view of the outcomes and would evaluate its reproducibility. Although MMSE and MOCA are screening tests, they were used herein for comparison with a continuous assessment tool, due to the mere lack of clinical assessment tests for continuous assessment of cognitive status. Their test–retest reliability did not allow for a higher granularity analysis of performance changes based on the participants’ cognitive abilities over time. Further, Breton et al. [44] have demonstrated that MMSE performs poorly in the detection of MCI, and has been discouraged as a comparison for new tests for MCI diagnosis. The different sample sizes of the groups may have affected the ability to detect differences between groups. In summary, we stress that this work was not intended to show the merit of FitForAll in the form it was presented in the paper, but rather to show the potential value of in-game metrics in carefully designed serious games. Our paper attempted to provide evidence for the value of the untapped assessment aspects of serious games such as FitForAll.

## 5. Conclusions

Our scope was to provide evidence that in-game metrics of SGs can have additional value. This piece of work reported on the implementation of stealth assessment in exergames targeting older adults. The results reveal evidence that careful design with respect to in-game metrics could potentially contribute to the early and unobtrusive detection of cognitive decline. Moreover, in line with the trend of researchers’ acceptance of SGs as new treatment options [13], additional research efforts should focus on providing sufficient evidence for the potential clinical value of SGs in terms of assessment [45]. Given the increasing number of studies published in the last few years demonstrating games as a complementary asset to classic and neuropsychological clinical tests, the importance of our findings and their potential to empower contemporary public health informatics and digital health is notable.

## Figures and Tables

**Table 1:** Weighted metrics used for scoring. More than one game contributes to the score of each domain.

Games (Domain)	Score Equation
Hiking and Cycling (Aerobic)	$\frac{\mathrm{Distance}\;\mathrm{Travelled}\;}{\mathrm{Total}\;\mathrm{Distance}\;\mathrm{To}\;\mathrm{Travel}\;\mathrm{in}\;a\;\mathrm{fixed}\;\mathrm{time}\;\mathrm{window}\;}$
Strength exercises (Strength)	$\frac{\#\mathrm{Correctly}\;\mathrm{performed}\;\mathrm{Iterations}}{\#\mathrm{Total}\;\mathrm{Iterations}}$
Stretching exercises (Flexibility)	$\frac{\#\mathrm{Correctly}\;\mathrm{performed}\;\mathrm{Iterations}}{\#\mathrm{Total}\;\mathrm{Iterations}}$
Steps (Balance)	$\frac{\#\mathrm{Correctly}\;\mathrm{performed}\;\mathrm{Iterations}}{\#\mathrm{Total}\;\mathrm{Iterations}}$
Apple (HRMG)	$0.8*FinishTime+0.2*\;\frac{\#ApplesGathered}{\#TotalApples}$
Arkanoid (HRMG)	$0.4*\;\frac{\#HitTargets}{\#TotalTargets}+0.6*\;\frac{\#RemainingLives}{\#TotalLivesAtStart}$
Fishing (HRMG)	$\frac{\#CaughtFish}{\#TotalFish}$
Golf (HRMG)	$0.7*\;\frac{DistanceTravelled}{OptimalPathwayDistance}+0.2*BallScored\;\left(True/False\right)+0.1*\;\frac{TimeToScore}{TotalTime}$
SkiJump (HRMG)	$\frac{DistanceTravelled}{MaximumPossibleDistance}$

**Table 2:** Description of group demographics and assessment tests score per cognitive group (cognitive groups according to the clinical diagnosis).

	Cognitively Normal (CN)	MCI	MD
#Participants	38	64	14
Females	30	54	11
Age (years)	67.1 ± 5.2	69.3 ± 6.4	77.7 ± 3.4
Education (years)	8.5 ± 2.6	7.6 ± 2.8	5.8 ± 4.3
MMSE	28.1 ± 1.2	26.5 ± 2.2	21.7 ± 1.5
MOCA	26.2 ± 2.4	22.43 ± 2.9	16.0 ± 2.3
TMT A	70.0 ± 32.3	86.9 ± 36.3	178.1 ± 90.4
TMT B	141.9 ± 64.1	189.7 ± 76.5	298.9 ± 80.1
Strength	7.6 ± 1.2	7.6 ± 0.9	6.5 ± 1.7
Aerobic	6.8 ± 1.6	6.4 ± 1.4	5.8 ± 1.7
HRMG	5.2 ± 1.2	4.7 ± 0.8	3.3 ± 0.7
Flexibility	8.7 ± 1.0	8.9 ± 0.4	8.3 ± 0.9
Heart Rate	74.0 ± 10.2	72.6 ± 9.8	72.0 ± 9.2
Borg Scale	6.9 ± 1.2	7.1 ± 1.2	7.2 ± 1.0

**Table 3:** Detailed accuracy for each cognitive clinical diagnosis when classifying among normal, MCI and MD (116 total instances).

Cognition	TP Rate	FP Rate	Sensitivity	Specificity	ROC Area
Cognitively Normal	0.684	0.179	68.4%	82.1%	0.785
MCI	0.734	0.308	73.4%	69.2%	0.734
MD	0.643	0.039	64.3%	96.1%	0.875

## Data Availability

The data presented in this study are available on request from the corresponding author. The data are not publicly available due to ethical reasons.
